# Inflammatory breast cancer tumor emboli express high levels of anti-apoptotic proteins: use of a quantitative high content and high-throughput 3D IBC spheroid assay to identify targeting strategies

**DOI:** 10.18632/oncotarget.15667

**Published:** 2017-02-24

**Authors:** Jay Arora, Scott J. Sauer, Michael Tarpley, Peter Vermeulen, Charlotte Rypens, Steven Van Laere, Kevin P. Williams, Gayathri R. Devi, Mark W. Dewhirst

**Affiliations:** ^1^ Duke Cancer Institute, Duke University, Durham, NC, USA; ^2^ Department of Surgery, Division of Surgical Sciences, Duke University, Durham, NC, USA; ^3^ Department of Radiation Oncology and Imaging Program, Duke University, Durham, NC, USA; ^4^ Trinity College of Arts and Sciences, Duke University, Durham, NC, USA; ^5^ Department of Pharmaceutical Sciences, Biomanufacturing Research Institute and Technology Enterprise, North Carolina Central University, Durham, NC, USA; ^6^ Translational Cancer Research Unit, Oncology Center, General Hospital Sint Augustinus, Center for Oncological Research (CORE), University of Antwerp, Antwerp, Wilrijk, Belgium

**Keywords:** NFκB, XIAP, disulfiram, oxidative stress, apoptosis

## Abstract

Inflammatory breast cancer (IBC) is one of the most lethal breast cancer variants; with existing therapy, 5-yr survival rate is only 35%. Current barriers to successful treatment of IBC include frequent infiltration and the presence of tumor cell clusters, termed tumor emboli, within the breast parenchyma and lymphatics. Prior studies have identified the role of anti-apoptotic signaling, in particular hyperactivation of NFκB and its target genes, in IBC pathobiology and therapeutic resistance. The objectives of this study were to: (1) determine if IBC tumor emboli express anti-apoptotic proteins and (2) develop a high content, multiparametric assay to assess the morphology of the IBC 3D spheroids and to optimize a high throughput format to screen for compounds that can inhibit the formation of the IBC tumor clusters/embolic structures. Immunohistochemical analysis of IBC patient tumor samples with documented tumor emboli revealed high NFκB (p65) staining along with expression of XIAP, a potent anti-apoptotic protein known to interact with NFκB signaling in enhancing survival of malignant cells. Subsequently, the high content assay developed allowed for simultaneous imaging and morphometric analysis, including count and viability of spheroids derived from SUM149, rSUM149 and SUM190 cells and its application to evaluate XIAP and NFκB inhibitory agents. We demonstrate the efficacy of the off-patent drug disulfiram when chelated with copper, which we had previously reported to inhibit NFκB signaling, was highly effective in disrupting both IBC spheroids and emboli grown *in vitro*. Taken together, these results identify a high-throughput approach to target tumor spheroid formation for drug discovery. Finally, disulfiram is a safe and approved drug for management of alcohol abuse, warranting its evaluation for repurposing in IBC therapy.

## INTRODUCTION

Inflammatory breast cancer (IBC) is considered the most lethal form of locally advanced breast cancer and although categorized as a rare disease, IBC accounts for more than 15% of breast cancer related deaths [1, 2]. IBC is a designated health disparity due to its increased incidence and mortality amongst minority populations both in the US and globally. With improved awareness, its detection has increased, especially in large clinical centers [3]. The majority of patients at the time of diagnosis are already in advanced Stage III or higher with lymph node involvement; 30% have distant metastases at the time of diagnosis [4, 5]. Although patients receive aggressive multidisciplinary treatments including anthracycline/taxane-based neo-adjuvant chemotherapy, targeted drugs, radical surgery, and adjuvant radiotherapy, nearly 50% of patients die of metastatic disease. It is therefore clear that IBC tumor cells develop mechanisms to evade therapeutic apoptosis leading to cancer recurrence. It has been challenging to identify molecular mechanisms that drive tumor aggression in patients with IBC. For example, all molecular subtypes associated with breast cancer are present in IBC [6]. Second, prognostic differences related to chromosomal alterations in IBC are also not significantly different from other advanced breast cancers [7].

Interestingly, the most distinct histopathological feature associated with all IBC patients is the presence of tightly packed tumor cell clusters (also termed tumor emboli) in the dermis tumor parenchyma, lymphatic vessels, and other metastatic sites [8]. Unlike other breast cancers, there is usually no solid mass or lump that can be felt during a breast exam or imaged on a mammogram. During metastatic progression, the tumor cell clusters appear to spread and block the lymph vessels in the skin of the breast, causing redness, swelling, and pain. The symptoms mimic inflammation (hence the name) and can often lead to misdiagnosis of an infection. Interestingly, unlike non-IBC tumors wherein epithelial markers are lost as part of the epithelial-mesenchymal transition (EMT), leading to single cell invasion and dissemination through blood vessels, IBC tumor cells seem to retain epithelial markers. These include high E-cadherin expression, while also gaining mesenchymal and stem-like (aldehyde dehydrogenase [ALDH]-positivity) characteristics [9, 10]. These observations indicate IBC cells exhibit a high degree of cellular plasticity that is postulated to protect IBC tumor clusters/emboli from cell death/stress stimuli during cancer progression and metastasis [11].

We were the first to discover high expression of the most potent anti-apoptotic protein, XIAP, in IBC preclinical models [12–14]. To identify this resistance mechanism we isolated and clonally expanded tumor cell isolates that gained the ability to grow despite inhibition of oncogenic signaling mediated by epidermal growth factor (EGFR/HER2) and downstream protein kinase (MAPK or AKT) signaling. This process was successful in basal-type and HER2-overexpressing IBC cells [13, 15]. A comprehensive expression analysis of the surviving cell isolates with elevated XIAP led to the identification of an adaptive stress response signature/metagene dominated by anti-apoptotic, antioxidant, and cell survival activity [16–18]. We then applied this metagene to the World IBC Consortium tumor samples [6] and identified that untreated tumor samples from IBC patients compared to non-IBC and normal breast samples have a heightened expression of this NFκB pathway enriched signature [19, 20]. Although prior such studies [7, 20–22] reveal that IBC tumors express gene profiles that promote a hyperproliferative phenotype, they lack information of the expression pattern in the tumor cells and in particular if anti-apoptotic factors like NFκB contribute to tumor emboli formation. However, due to the diffuse nature of the disease, it is very difficult to obtain tissue with adequate numbers of dermal tumor emboli for detailed analyses. An alternative would be to grow tumor spheroids and emboli *in vitro*, using IBC cell lines. Tumor spheroids in culture generated from various cancer cell lines including breast, ovarian, liver, and colon cancer are widely used as 3D models grown in non-adherent conditions [23–25]. In this study, we optimized a high content multiparametric assay to simultaneously image and quantitatively measure multiple cell health characteristics of 3D IBC tumor spheroids. This method was then applied to characterize the efficacy of various candidate anti-cancer compounds that target the XIAP:NFκB survival pathway. The study found that an FDA-approved drug used over fifty years for chronic alcoholism, disulfiram (DSF), when chelated with copper suppressed IBC tumor emboli formation. Furthermore, NFκB and XIAP expression were observed to be significantly higher in tumor embolic structures in IBC patient tumor tissue. These results revealed the potential of using the NFκB inhibitor, DSF in combination with copper for treatment of IBC.

## RESULTS

### High NFκB and XIAP expression in IBC tumor emboli

**Figure 1:**
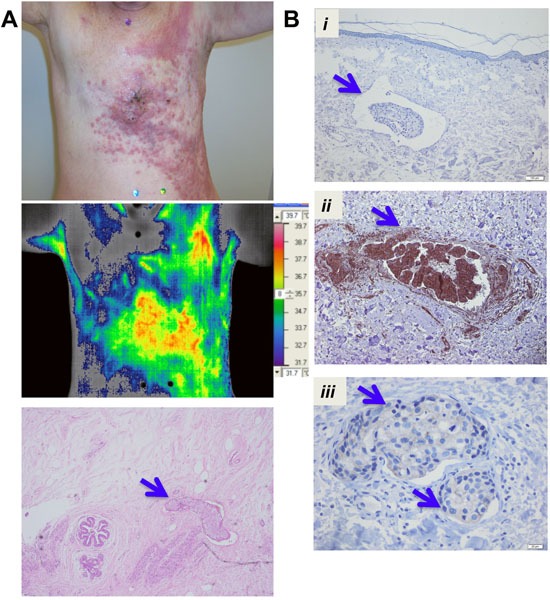
XIAP and NFκB expression in tumor emboli in IBC patient samples **A**. Representative photographic images to show the diffuse presence of tumor cell clusters of a de-identified patient with chest wall recurrence including thermogram of temperature, a potential indicator of elevated blood flow and metabolic activity in tumor cells measured using infrared thermography (scale bar shown). H&E staining of a representative tumor section showing an embolic structure in the lymphatic vessels (blue arrow). **B**. Immunohistochemistry analysis showing tumor emboli (blue arrows) identified in samples of pretreatment IBC primary tumor tissues and representative images from i. Negative control, ii. NFκB; iii. XIAP stained sections.

The diffuse nature of the disease with tumor cells clustered all over the chest wall is revealed in a representative photographic image of a patient with tumor recurrence. Furthermore, the associated thermogram showing elevated temperature in the tumor lesions likely reflects relatively higher perfusion, combined with an elevated metabolic rate, compared with surrounding tissue and overall reveals the aggressive nature of this breast cancer subtype (Figure 1A). Representative H&E staining shows the presence of tumor emboli in the tumor tissue (Figure 1A, bottom panel). To address if high expression of anti-apoptotic proteins contributes to the aggressive phenotype in IBC, we evaluated the expression of XIAP and NFκB, key pro-survival factors, in tumor emboli present in untreated IBC patient tumor samples made available through the World IBC Consortium from a total of 14 samples with documented tumor emboli as described in methods. Immunohistochemical analysis of the tumor sections revealed significant expression of NFκB (p65 subunit) in all the identified tumor embolic structures and XIAP expression in 8/14 samples with ranging degree of staining (Figure 1B). Therefore, these results identifying expression of NFκB and XIAP proteins that are known to crosstalk and regulate anti-apoptotic signaling in cancer cells suggest the potential of targeting these survival proteins for inhibiting tumor clusters/emboli formation, postulated to be one of the first steps in IBC progression and metastatic dissemination.

### Development and optimization of a high content, high throughput assay for quantitation of phenotypic markers in 3D IBC cell clusters grown *in vitro*

**Figure 2:**
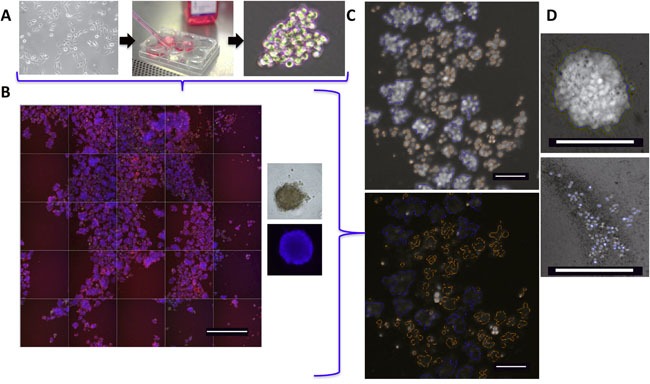
IBC 3D tumor spheroid high content assay **A**. Schema of IBC tumor spheroid culture in ultra-low attachment plates and analysis after addition of specific dyes. **B**. Representative montage of 25 neighboring fields from a single well (10x – scale bar = 800 microns) of spheroids stained with Hoechst 33342, YOYO-1 and MitoTracker Red FM dyes. Right - Enlarged image of a single SUM149 tumor cell cluster shown (phase contrast and Hoechst stained). **C**. Representative image analysis (10x – scale bar = 100 microns) of SUM149 spheroids at day 6, with masks overlaid over the spheroids (top) or masks alone (bottom) established by Hoechst signal and a >50 μm area cutoff. These regions of interest are then used for analysis of spheroid YOYO-1 and MitoTracker staining. **D**. Image of a single spheroid (top - untreated and bottom - treated with a cell death inducing agent) to highlight the spheroid mask (blue outline) and morphology.

In order to study if targeting the anti-apoptotic proteins can inhibit the ability of IBC cells to form tumor cell clusters and subsequent viability, we employed IBC patient primary tumor derived lines, SUM149 (triple negative, EGFR activation) and SUM190 (estrogen receptor negative, HER2 overexpression). These cells have the ability to spontaneously form 3D tumor spheroids when seeded in ultra-low attachment plates similar to previous studies [19, 27, 30]. Representative images of SUM149 tumor spheroids are shown in in Figure 2A. We then applied a high content “Cell Health Profiling” assay to the IBC tumor spheroids by using three dyes, Hoechst 33342 (to measure spheroid count and morphology), YOYO-1 (infiltrates cells with compromised membranes and quantifies viability) and MitoTracker Red FM (determines mitochondrial membrane potential). This strategy allowed us to image the formation and changes in size and number of resultant IBC cell clusters/spheroids over time across multiple wells (Figure 2B). This offers the advantages of simultaneously providing qualitative images, numeric measures of cell health characteristics and growth kinetics of intact tumor cell clusters/spheroids, which can also be grown in multi-well plates for high throughput screening. Data in Figure 2C is a representative view of a field with multiple spheroids while Figure 2D shows a representative single spheroid (untreated, healthy [upper], and treated with a cytotoxic agent [lower]). In general, using the Hoechst signal we included spheroids of > 50 μm diameter as sizes smaller may reflect cell aggregates (Figure 2B- orange spheroid masks were excluded while blue masks indicate included spheroids). The masking also facilitates determination of various characteristics of the multicellular tumor spheroids (Table 1) as well as establishing the regions of interest for quantitation of YOYO-1 (area inside nuclear mask; channel 2 mean average intensity) and MitoTracker Red FM staining.

**Table 1:** Multiparametric cell health measurements of the multicellular tumor spheroids derived from IBC cells grown in low-attachment plate analyzed in the high content assay

Dye	Emission Color (Wavelength)	Measurement	Cellular Determinant	Spheroid/Individual Cell Parameter
Hoechst 33342	Blue (386 nm)	Mean_Object Size Mean_Object Shape LWR Mean_Object Shape P2A Mean_Object Variable Intensity Ch1	Size Nuclear Aspect Ratio Roundness (Perfect circle = 1) Texture	**Morphology**
Hoechst 33342	Blue (386 nm)	Object count/Field	Spheroid density per valid field	**Proliferation**
YOYO-1	Green (485 nm)	Mean_Target Average Intensity, Ch 2 (*YOYO-1 pixel intensity normalized to spheroid size)*	Plasma membrane integrity	**Viability**
Mitotracker FM Red	Red (549 nm)	Mean_Target Average Intensity, Ch 3 Mean_Target Total Intensity, Ch 3 *(Mitotracker pixel intensity normalized to spheroid size)*	Average brightness of functional mitochondria Total mass of functional mitochondria	**Mitochondrial Function/Membrane Potential**

**Figure 3:**
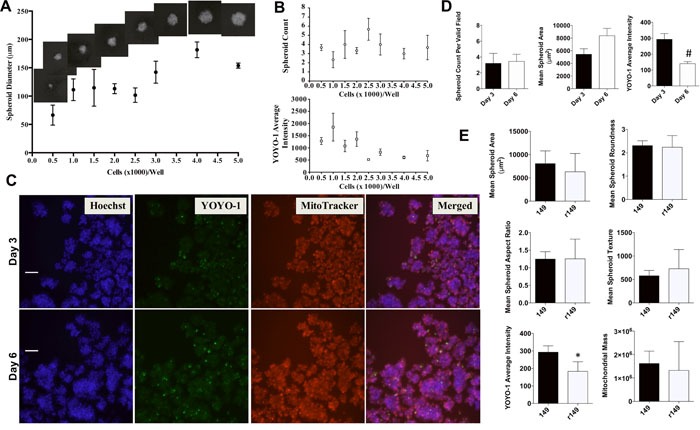
Assessment of SUM149-derived tumor spheroid morphology **A**. SUM149 cells at different seeding densities and visualized on day 3 to assess 3D spheroid size, Representative spheroid images (10x) inset above Figure 3A to show the change in spheroid size for each seeding density. **B**. Spheroid viability as quantified by average YOYO-1 intensity (top) and count (bottom). Representative data shows mean ± SEM for each parameter [replicates = 3]. **C**. Representative pictures of individual channel signal and merged overlay showing SUM149 tumor spheroids at days 3 and 6 stained for nuclei (Hoechst), viability (YOYO-1) and mitochondrial membrane potential (MitoTracker Red). Cells fixed before imaging. 150-175 fields of view imaged for each well and 500 fields of view per dye for each well at 10x magnification (Scale bar = 100 microns). **D**. Quantitative analysis of SUM149 tumor spheroids at day 3 and day 6 for number of spheroids, area (μm^2^), viability measured by YOYO-1 average intensity. Bars represent mean ± SEM for each parameter [replicate fields/well = 5-18; #*P*<0.005 comparisons made between days 3 and 6]. **E**. Quantitative analysis of drug resistant rSUM149 cell line (r149)-derived tumor spheroids compared with SUM149 (149)-derived tumor spheroids at day 3 for indicated parameters on the Y axis in each graph, which includes area (μm^2^), roundness (shape P2A), aspect ratio (shape LWR), texture (Hoechst variable intensity), YOYO-1 average intensity, mitochondrial mass (total MitoTracker intensity), and mitochondrial brightness (average MitoTracker intensity). Bars represent mean ± SEM for each parameter [replicate fields of viable spheroids/well = 6; *P<0.05 comparisons made between SUM149 and rSUM149].

The IBC spheroid formation was then assessed for varying seeding densities over multiple days in culture (Figure 3). An increase in spheroid size is observed with increasing cell seeding density (Figure 3A), with no significant change in total number of spheroids (Figure 3B). Average YOYO-1 intensity is variable until seeding densities ≥ 2500 cells/well, when the spheroids approach their maximum size and the YOYO-1 signal is stable and low, suggesting healthy mature spheroids (Figure 3B – top). Overall, these measurements were used to optimize seeding densities for different plate sizes as described in Materials and Methods. Representative images are shown in Figure 3C highlighting individual spheroid staining and a merged overlay of all dyes staining the tumor spheroids at days 3 and 6 in a 6-well plate. We observed uniform Hoechst and MitoTracker staining within the tumor cells in the untreated 3D spheroids, although the periphery of some spheroids stained more intensely with MitoTracker. In addition, the untreated spheroids had minimal YOYO-1 staining and therefore reflect the formation of viable tumor spheroids in this assay. The acquired images at the early (day 3) and later time points (day 6) were used to derive multiparametric phenotypic and viability quantitative measures, which are summarized in Table 1 and Figure 3D. Spheroid area increased over time saturating in size after day 7, with no change in spheroid number after day 3. Certain parameters in this assay were normalized by determining mean staining per object to account for differences in spheroid size (YOYO-1, MitoTracker Red). It is to be noted that the numerical values associated with measurements of spheroid morphology or mitochondrial transmembrane potential can show increases or decreases in signal relative to untreated spheroids, both of which can be indicative of compromised cell health or spheroid integrity [31].

Next, we tested the spheroid-forming ability of the drug resistant variant rSUM149 that has been previously shown to correlate with high endogenous XIAP and NFκB expression and decreased sensitivity to therapeutic apoptosis [13, 18]. The rSUM149 cells also formed tumor spheroids within 24-48h in non-adherent culture conditions. Quantitative analysis of the multicellular spheroid morphology (area, shape, texture), YOYO-1 intensity (cell viability within the spheroids) and MitoTracker Red staining (mitochondrial membrane potential of the cells within the tumor spheroids) after 3 days of growth is summarized in Figure 3E. Although there was some variation in rate of spheroid formation between the parental and drug resistant lines (data not shown), there were no statistical difference observed in the spheroid area, morphological parameters or mitochondrial membrane potential between SUM149 and rSUM149 cells. These results reveal the potential of using these cell lines in parallel for drug screening studies of spheroids formed from both therapy-sensitive and -resistant isotype-matched variants.

### Targeting NFκB disrupts *in vitro* IBC tumor spheroid formation

**Figure 4:**
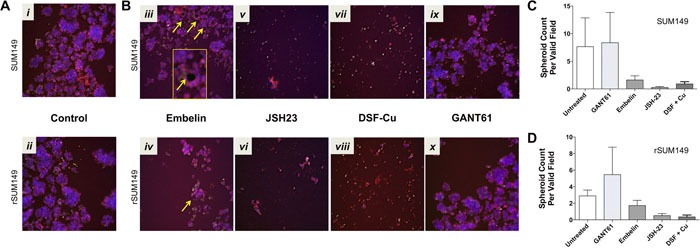
Application of 3D high content assay to test compounds known to target XIAP and NFκB pathways in parental SUM149 and isotype matched drug-resistant isolate, rSUM149, cells Representative images (merged overlay of all channels, 10x – scale bar = 100 microns) from spheroids derived from SUM149 and rSUM149 cells **A**. DMSO treated controls (*i* – SUM149*, ii* – rSUM149); **B**. treated with inhibitors that target XIAP (25 μM embelin [*iii* – SUM149*, iv* – rSUM149]), NFκB (100 μM JSH-23 [*v* – SUM149*, vi –* rSUM149] or 100nM DSF in combination with copper [*vii* – SUM149*, viii* – rSUM149)] or GLI1 as a non-targeting control (10 μM GANT61[*xi* – SUM149*, x* – rSUM149]). Quantitative graphical analysis of spheroid numbers derived from SUM149 **C**. and rSUM149 **D**.

**Figure 5:**
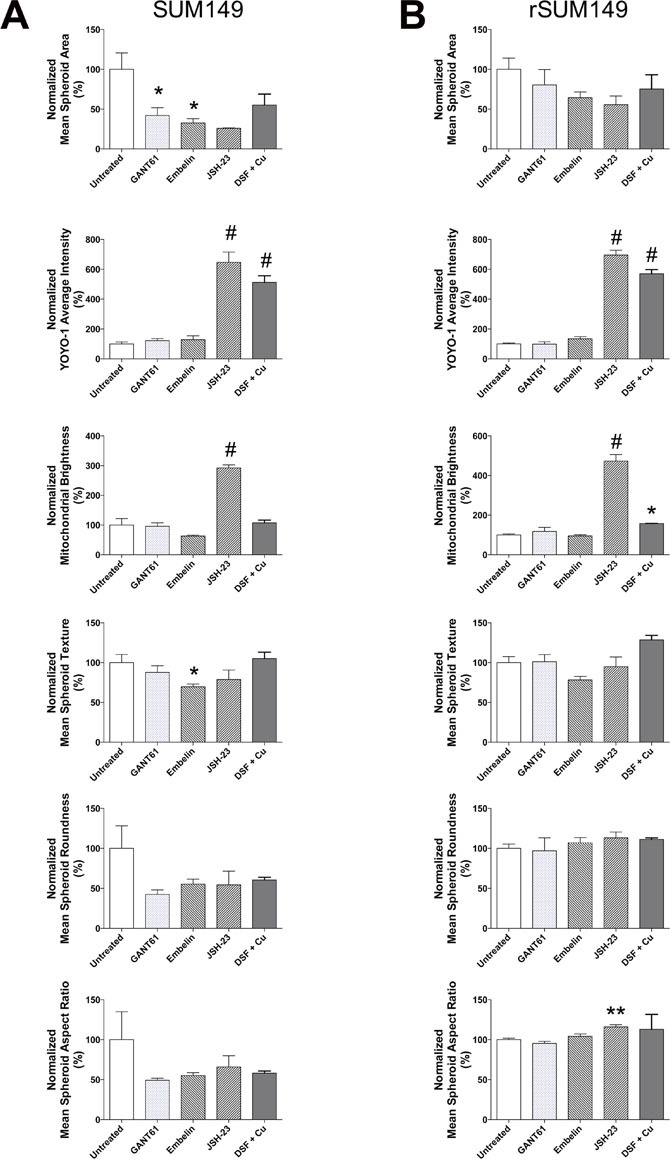
Quantitative analysis of XIAP and NFκB inhibitors on IBC tumor spheroid cell morphology, viability and mitochondrial function **A**. SUM149 and **B**. rSUM149 tumor spheroids at day 3 after indicated treatments. Bars represent mean ± SEM for each parameter [replicate fields of viable spheroids/well = 2-11; *P<0.05, **P<0.01, #*P*<0.005 comparisons made between each treatment and untreated].

Based on our aforementioned studies in patient tumor samples showing high levels of NFκB and XIAP in tumor embolic structures, we wanted to test if targeting the XIAP-NFκB anti-apoptotic signaling pathway can inhibit formation of tumor cell clusters and/or induce cell death using the high content IBC 3D spheroid assay developed herein. To provide proof of principle, we evaluated known XIAP and NFκB pharmacological inhibitors, which in our prior studies have also shown their efficacy in SUM149, rSUM149 and SUM190 IBC cell lines grown in 2D cultures. These included embelin (a plant derived non-peptidomimetic that targets XIAP's ability to inhibit caspase 9 and inhibits NFκB activity) [17], JSH23 (inhibits nuclear localization of NFκB) [18], and GANT61, a hedgehog/Gli1 inhibitor linked to anti-apoptotic signaling in IBC cells [32]. The compounds were added during cell seeding to test their ability to disrupt the formation and growth of spheroids during the 3-6 day period in non-adherent culture. Representative images from the high content analysis are shown in Figure 4 and the numeric data from multiple such high content imaging experiments were analyzed for quantitative measures as elaborated in Table 1 for the treatment effects in both SUM149 (Figure 4A, 4C) and rSUM149 cells (Figure 4B, 4D). Figure 5 details the multiparametric analysis of the treated spheroids. Results show that compared to untreated and DMSO (vehicle) treated cells, the NFκB inhibitor, JSH23, was highly efficacious in disrupting formation of both SUM149 and drug-resistant rSUM149 tumor spheroids in culture (Figure 4B – v [SUM149] and vi [rSUM149]). JSH-23 treatment significantly increased average YOYO-1 staining in both sensitive and resistant cell lines as well as mitochondrial brightness, indicative of decreased cell health (Figure 5A & 5B). In contrast, cells treated with the XIAP antagonist, embelin did not completely inhibit formation of tumor spheroids in the case of SUM149 cells (Figure 4B – iii [SUM149] and iv [rSUM149]). However, embelin-treated IBC tumor spheroids exhibited multiple empty/vacuole-like regions (shown by yellow arrows in Figure 4) in the core compared to control spheroids and a 70% decrease in spheroid area, 40% lower mitochondrial membrane potential, and a 30-45% change in texture, shape, and aspect ratio in SUM149 spheroids. Low YOYO-1 staining supports the live-imaging observations (Figure 5) that embelin does not significantly inhibit tumor spheroid formation in either cell line. GANT61, which does not target the XIAP-NFκB axis, did not have an appreciable effect on tumor spheroid morphology in either cell lines at the concentration reported previously to be effective in 2D cultures, relative to untreated and DMSO controls, although certain parameters like spheroid area, spheroid aspect ratio and roundness were affected with treatment. None of the agents tested have a significant impact on spheroid morphology (texture, roundness, aspect ratio).

These results collectively show that quantitative analysis of the images captured in these experiments included the derivation of multiple parameters that allow for the assessment of morphological features of 3D IBC tumor spheroids, as well as viability and reveal that the tumor spheroid-HCA assay may be suitable for high-throughput, multiparametric, anticancer drug screening.

### Disulfiram in combination with copper disrupts formation of IBC tumor spheroids and emboli grown *in vitro*

Recently, we reported that the FDA-approved drug disulfiram when combined with copper (DSF-Cu) enhances uptake in cancer cells and inhibits pNFκB leading to decreased XIAP, enhanced oxidative stress-induced apoptosis and significant inhibition of SUM149 tumor growth in a murine xenograft model [19]. Based on this we tested if the same DSF concentrations (100 nM and 300 nM) in combination with copper chelation would inhibit formation and viability of IBC tumor cell clusters. The 3D high content IBC tumor spheroid assay showed that compared to untreated, DSF combined with copper was associated with extensive cell death (low spheroid number and high YOYO-1 staining) in both the parental SUM149 and the drug/apoptosis resistant rSUM149 cells (Figure 4B-vii and 4C [SUM149], 4B-viii and 4D [rSUM149] & Figure 5).

**Figure 6:**
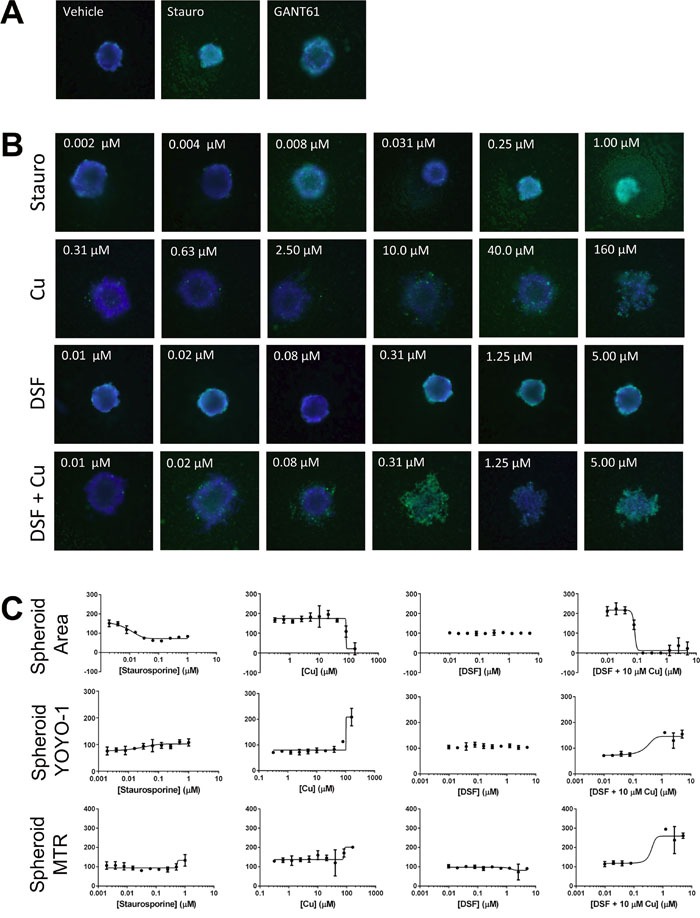
DSF in combination with copper inhibits SUM149-derived spheroids grown in a high-throughput assay format SUM149 cells plated at 400 cells/well in a 384-well ultra low attachment plate treated with indicated compounds and includes a 10-pt dose response, 1:2 dilutions of Staurosporine (0.002 μM – 1 μM); CuSO_4_ (0.3 μM – 160 μM), DSF alone (0.01 μM – 5 μM); and combination of DSF (0.01 μM – 5 μM) + 10 μM CuSO_4_ (DSF+Cu) and single concentration of GANT 61 (10 μM). Representative images **A., B**. of Hoechst and YOYO-1 channels merged for a combined assessment of spheroid area, integrity and growth inhibition are shown along with **C**. graphical presentation of the quantitative multiparametric analysis.

**Figure 7:**
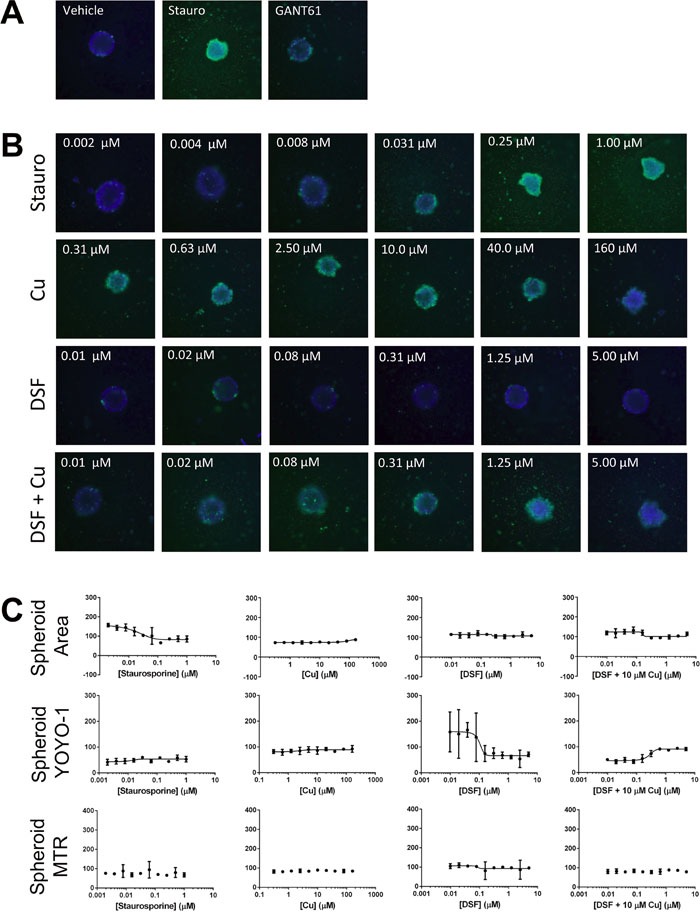
Targeting formation of SUM190-derived spheroids in the high content, high-throughput assay High content analysis reflects changes in SUM190-derived spheroid area and spheroid integrity after cells (800 cells/well of a 384 well plate) treated with indicated compounds (10-pt dose response, 1:2 dilutions of treatment similar to the ones presented in Figure 6). Representative images **A., B**. of Hoechst and YOYO-1 channels merged for a combined assessment of spheroid area, integrity and growth inhibition are shown along with **C**. graphical presentation of the quantitative multiparametric analysis.

To facilitate testing of extended dose responses of compounds in multiple cell lines we optimized the high content tumor spheroid assay for high throughput in ultra-low attachment 384-well plates (Supplementary Figure 1) with the result that one spheroid forms per well as explained in Methods. Compounds were added in 2-fold 10 point dose response in replicates of five and subsequently stained with Hoechst 33342, YOYO-1 and Mitotracker red dyes at 72h to assess spheroid area, spheroid integrity, and active mitochondrial content respectively. We tested the effects of DSF with and without copper on two IBC cell lines SUM149 (Figure 6) and SUM190 (Figure 7). In both cell lines, staurosporine, a potent cell death agent, was effective at reducing spheroid area with a concomitant increase in YOYO-1 staining reflecting spheroid disruption. DSF alone at all concentrations tested, including the 300 nM dose, had minimal affect on spheroid growth for both SUM149 and SUM190 cells. As another control we also tested a dose range of copper alone. As expected at the 10 μM dose there was no affect on spheroid area and only an affect seen at very high non-physiological does (160 μM). These studies further confirmed the effect of the combination of DSF with Cu to be highly effective at disrupting tumor spheroids in SUM149 cells as assessed by decreased spheroid area (IC_50_ = 0.08 μM) and integrity (increasing YOYO-1 IC_50_ = 0.2 μM). An approximately 2-fold increase in MitoTracker staining was also seen for SUM149 cells when treated with DSF + Cu above 1μM. GANT61, a hedgehog pathway inhibitor had minimal effects on spheroid growth at the concentration tested and served as a surrogate pathway control as it is not recognized as a compound that inhibits XIAP or NFκB expression. Furthermore, we investigated tumor spheroids cultured from another patient primary tumor derived IBC cell line, HER2 overexpressing SUM190 cells. SUM190 spheroid had about 45% decrease in area with increasing staurosporine concentration (EC_50_ = 0.02 μM) and YOYO-1 increases with both staurosporine and DSF + Cu treatment, however, the data show that DSF-Cu treatment was less potent when compared to its effect in the triple negative, EGFR activated SUM149-derived spheroids.

**Figure 8:**
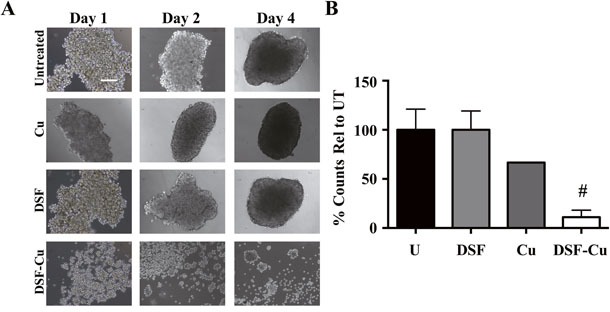
DSF-Cu disrupts formation of IBC tumor emboli under *in vitro* simulated growth conditions **A**. Representative images (10 x – scale bar = 200 microns) of SUM149 cells grown under tumor emboli conditions for 1-4 days and treated with 10 μM copper (Cu), 100 nM disulfiram (DSF) or DSF-Cu. **B**. Percent of spheroid number relative to untreated for SUM149 cells grown for 4-5 days [#*P*<0.005 comparisons made between treatment and untreated].

Although most breast cancer cell lines form 3D spheroids/mammospheres when grown in ultra-low attachment conditions with spheroid-promoting media, IBC tumor cell clusters/emboli are distinct due to their unique presence and metastasis in the lymphatic system. To study this, we used a recently reported method [27] wherein IBC cells are cultured in media supplemented with PEG-8000 and grown on an orbital shaker, simulating the viscosity and shear force of the lymphatic system. These conditions allow IBC cells to form tight tumor cell clusters with similar phenotype and histological aspects observed in tumor emboli found in patient samples. Data in Figure 8 show formation of SUM149-derived embolic structures *in vitro* over indicated time points and the effect of compounds tested. SUM149 tumor emboli were significantly inhibited by 100 nM DSF+10 μM Cu, with an 89% reduction (*P*<0.005) in spheroid number relative to untreated and most cells observed to be dispersed single cells compared to DSF and copper alone. Taken together, these results reveal the efficacy of DSF-Cu in targeting the formation of IBC tumor clusters in culture using two different assay conditions.

## DISCUSSION

The key role of NFκB-associated pathways in IBC compared to other stage- and subtype-matched advanced breast cancers is based on three major lines of evidence. First, identification of NFκB target genes involved in the proliferation, inflammation and invasive processes that are dysregulated at the clinical and molecular level in IBC. Second, analysis of IBC tumors showing abnormal expression of NFκB target genes involved in adaptive stress responses that confer a death resistance phenotype in cancer cells. Third, stress-mediated translational induction of the anti-apoptotic signaling protein, XIAP, corresponds with multi-drug resistance. The present study is the first to identify that tumor emboli observed in IBC patient tumor tissue express high levels of NFκB and XIAP. The functional implication of the tumor emboli in IBC patients can be envisioned to - 1) obstruct the draining lymph vessels and explain the clinical feature of breast swelling, chest wall inflammation and extreme pain; 2) be a key step that drives the local spread of cancer cells throughout the breast gland through self-seeding and invasion. Therefore, there is a critical need to elucidate dysregulated pathways that promote IBC tumor emboli formation in order to design improved targeted therapeutics.

Due to the diffuse nature of IBC disease, it is extremely difficult to obtain tissue with adequate numbers of dermal tumor emboli and therefore, there is a need for improved *in vitro* approaches. Multicellular culture systems, in particular *in vitro* 3D spheroid models of cancer cells, have shown wide applicability in preclinical drug screening studies; however, there is continued need to improve morphometric analysis of tumor cells comprising the spheroids and to customize this analysis for different cancer types. In the current study, we have optimized a high content assay that is amenable to medium- to high-throughput using a single-step multi-dye staining protocol to simultaneously characterize and quantitate multiple cell health characteristics of 3D IBC tumor spheroids both basally and after treatment with candidate compounds that can target XIAP and NFκB pathways. The results of the present study identified that NFκB inhibitors were potent in their ability to disrupt tumor spheroid formation resulting in decreased viability and changes in mitochondrial membrane potential during IBC tumor spheroid formation. Compared to direct inhibition of NFκB activity, embelin, which potentiates apoptosis by relieving the inhibitory effect of XIAP on caspase 9 activity caused some increase in cell death, in particular in the interior of the spheroids although the tumor spheroid formation itself was not inhibited. We then extended this method to multiple IBC cell lines, SUM149, SUM190 and rSUM149, which is an isotype-matched therapeutically resistant cell line derived from SUM149. Many of the parameters tested exhibited similar results between the lines although some dye-related differences were noted. YOYO-1 staining was slightly different between these cell lines (Figure 3E), possibly due to the higher levels of the survival proteins like NFκB and XIAP in rSUM149 cells relative to their parental counterparts [15]. Additionally, we observed that some untreated spheroids tended to have higher MitoTracker staining in the periphery relative to the center of the spheroids. While this may be a physiological phenomenon based on greater mitochondrial disruption on the periphery of the spheroids, the increased thickness in the center of the spheroids can lead to more light absorption and needs to addressed using other dyes and confocal imaging. The center of the spheroids may also be hypoxic, which would reduce mitochondrial activity [33] and we intend to investigate this possibility.

In addition to providing proof of principle for assay robustness, our studies identified the efficacy of a FDA-approved drug disulfiram, which when combined with Cu (DSF-Cu) has the ability to disrupt tumor spheroid formation. Herein, we also observed efficacy of DSF-Cu in disrupting emboli formation in a biologically relevant culture model reported to mimic the physical properties and environment of the dermal lymphatic system such that only IBC cells like SUM149 and SUM190 compared to other molecular-subtype matched non-IBC cells lines form the 3D structures when grown under these culture conditions [27]. Use of DSF-Cu as a therapeutic strategy is gaining momentum based on recent studies in various cancer types revealing the ability to target tumor cells while having minimal effect on normal cells [34]. Our previous study identified that in IBC cells, DSF-Cu increases copper concentration *in vivo*, bypassing the need for membrane transporters and this leads to increased oxidative stress mediated cell death in IBC tumor cells that are highly redox adapted [19]. Therefore, mechanisms of oxidative stress signaling in IBC tumor emboli, both *in vitro* and *in vivo*, warrant further investigation. Further development of the high content 3D IBC assay will increase the utility of this *in vitro* system for screening of potential anticancer compound libraries, combinations of drug modalities, and the effect of environmental chemicals. Additionally, the combination of disulfiram+copper should be tested in patient-derived xenograft (PDX) *in vivo* models of IBC, which will provide a method most closely modeling the human disease to assure disruption of IBC emboli in a living model [35].

In summary, the present observations show high NFκB expression along with its pro-survival signaling partner, XIAP, in the tumor emboli of IBC patient tumor tissue and efficacy of NFκB inhibitors like DSF-Cu in preventing formation of IBC tumor cell clusters/spheroid and emboli formation, considered a key feature of IBC progression and dissemination. These results, along with our previous report showing the ability of DSF-Cu to inhibit *in vivo* SUM149 tumor growth, strengthen the potential interest in pursuing DSF-Cu as a candidate for clinical trials in IBC patients.

## MATERIALS AND METHODS

### Cell lines and materials

SUM149 and SUM190 were obtained from Asterand, Inc. (Detroit, MI) and cultured as described previously [12]. Characterization and authentication of the cell lines were done at Asterand by short tandem repeat (STR) polymorphism analysis. rSUM149 is an isotype-matched drug resistant variant as previously described [13, 15] and cultured in SUM149 media supplemented with 7.5 μM of a research-grade lapatinib analog GW583340 (Tocris, Minneapolis, MN) 24h after splitting. All the cell lines are also authenticated at regular intervals by STR polymorphism analysis by the Duke DNA sequencing core. Cells were cultured in growth medium at 37°C under an atmosphere of 5% CO_2_, were banked upon receipt and cultured for no more than 6 months prior to use in this study. CuSO_4_ pentahydrate was purchased from VWR (Radnor, PA) and GANT61, embelin, JSH-23, DSF from Sigma-Aldrich (St. Louis, MO).

### Immunohistochemistry

Antibodies for immunohistochemical staining of p65 NFκB (clone C-20) and XIAP were purchased from Santa Cruz Biotechnology (Santa Cruz, CA) and BD Biosciences (San Jose, CA), respectively. Tissue sections stained for p65 NFκB were used as previously described [26]. Archived tissue sections from the World IBC consortium set with prior patient consent and approval from the Institutional Review Boards from each center obtained [6] were deparaffinized in a series of xylenes, then rehydrated in 95%, 85%, 70% ethanol and washed in distilled water and Dako wash buffer (Dako North America, Inc. Carpinteria, CA) for 5 min. Antigen retrieval was performed using citrate buffer pH6 incubated at 95°C for 35 minutes. After cooling and washing in Dako wash buffer, the slides were incubated with Dako peroxidase block for 5 min at room temperature. After washing 3 × 5 min in wash buffer, slides were incubated overnight at 4°C with the mouse anti-human XIAP antibody solution (1:60 dilution). After incubation, slides were washed 3 × 5 min with Dako wash buffer, then incubated in the anti-mouse secondary solution (provided in the Dako anti-mouse Envision kit) for 30 min at RT. To visualize antibody binding, slides were incubated with DAB+ substrate (Dako) for 10 min at room temperature. Finally, tissue sections were incubated in hematoxylin (Dako) for 30 seconds, washed in tap water and mounted for light microscopy. Prostate cancer tissue was used as positive control. A board-certified surgical pathologist scored the slides in a blinded manner. Staining intensity was graded on a qualitative scale (no staining [negative], very focal or very weak staining [borderline], and positive) and overall, the data were dichotomized as negative or positive (including borderline).

### *In vitro* propagation of 3D IBC tumor spheroids and emboli

SUM149 and rSUM149 cells were plated in 6-well (40,000 cells/well), 24-well (10,000 cells/well) ultra-low attachment, or 96-well (500-5,000 cells/well) black walled, round-bottom optical plastic ultra-low attachment plates (Corning, Corning, NY) using serum-free minimum essential media (MEM) (Invitrogen) supplemented with 20 ng/mL basic FGF, 20 ng/mL EGF and B27 (1x Invitrogen) for tumor spheroids and using Ham's F12 media (as explained in Cell Lines and Materials for the culture of SUM149 cells) additionally supplemented with 2.25% PEG-8000 as previously described [27] for tumor emboli. For high-throughput spheroid applications, SUM149 (400 cells/well) and SUM190 (800 cells/well) cells were plated in 384-well black walled, round-bottom optical plastic ultra low attachment plates (Corning, Corning, NY), centrifuged at 150 x g for 2 minutes and placed in a tissue culture incubator overnight. For low-throughput experiments, treatments were carried out at the time of seeding, and spheroids were grown for up to 8 days as indicated. 384-well plates for SUM149 and SUM190 cells were treated 1 day after plating utilizing a D300 digital compound dispenser (Hewlett Packard, Palo Alto, CA). Stock solutions of embelin (100 mM) [17], GANT61 (10 mM) [28], JSH-23 (10 mM) [29], and DSF made in dimethyl sulfoxide (DMSO) and subsequently diluted in media to concentrations as indicated, with concentrations based on previously published data regarding cytotoxicity of the compounds in SUM149 cells [15]. The final DMSO concentration of these solutions ranged between 0.03-1%.

### Tumor spheroid high content, high throughput multiparametric assay

To each well of the IBC tumor spheroids in 6-well, 24-well, 96-well plates, or 384-well plates, 1 mL, 250 μL, 50 μL, or 25 μL per well of pre-warmed 3x dye cocktail in PBS (30 μg/mL Hoechst 33342, 300 nM YOYO-1, 1.5 μM MitoTracker Red FM) was added, respectively. For the 96- and 384-well plates, the cells are plated using a Multidrop bulk dispenser and compounds added the following day using a Hewlett Packard D300 Digital Dispenser. Plates were then returned to the incubator for 1h followed by fixation with 1 mL (6-well), 250 μL (24-well), 50 μL (96-well), or 30 μL (384-well) of 10% formalin. Plates were then imaged immediately, or sealed, protected from light and stored at 4°C overnight. Initial imaging was conducted using a 4x objective for 96- or 384-well plates and 10x objective for 6- and 24-well plates (96- and 384-well round-bottomed plates were centrifuged for 1 min at 150 x g prior to imaging in order to center spheroid in the well). Fluorescence quantification and localization was determined using a ThermoFisher CellInsight NXT and 3-channel Cell Health Profiling protocol in HCS Screen software (ThermoFisher). Excitation wavelengths used were 386 nm, 485 nm, and 549 nm for Hoechst 33342, YOYO-1, and MitoTracker Red FM, respectively. Fixed exposure times were optimized in each channel for each experiment and set so that camera pixel intensity saturation was not reached. Images were then acquired using an Olympus UPlanFLN 10X/0.30 objective for 6- and 24-well plates or an Olympus UPlanSApo 4X/0.16 objective for round-bottom 96- and 384-well plates. All conditions were conducted in triplicate for low-throughput and quintuplicate for 384-well plate imaging (Supplementary Figure 1).

A mask was established by the channel 1 signal (Hoechst 33342) and used to determine general spheroid characteristics (size, area, aspect ratio, roundness, and texture) as well as to establish the regions of interest for YOYO-1 and MitoTracker Red FM staining (area inside channel 1 spheroid mask). YOYO-1 signaling was calculated using channel 2 mean or total average intensity and MitoTracker Red FM signaling was calculated using channel 3 mean target total intensity for mitochondrial mass and channel 3 mean target average intensity for mitochondrial brightness. Data analysis was performed after retrieving data sets from HCS View software (ThermoFisher) and normalizing to mean DMSO values. Image montages were generated for 6- and 24-well plates with data analysis applied only to objects identified entirely within one field of view. Spheroids in the multi-well round-bottom low attachment plates were centered in one field of view and analyzed accordingly. Spheroids out of focus or not entirely contained in the single field of view were excluded from analysis.

### Microscopy

Phase contrast microscope images (4X or 10X) were taken daily up to 6 days to assess 3D spheroid formation using a Motic AE2000 microscope (Motic in North America, Richmond, BC, Canada), M14 camera, and Infinity Capture (Lumenera, Ottawa, Canada) software.

### Statistical analysis

Quantitative data were expressed as mean ± SEM. Spheroid diameter was determined from spheroid area generated by automated imaging analysis [diameter = 2 x √(area/π)]. The statistical analyses were conducted using Graphpad Prism (Graphpad Software, Inc.) student's 2-tailed t-test, one-way Anova - Fisher's LSD test and differences were considered significant at p < 0.05.

## SUPPLEMENTARY FIGURE


