# Supplementary material for: Inflammatory breast cancer tumor emboli express high levels of anti-apoptotic proteins: use of a quantitative high content and high-throughput 3D IBC spheroid assay to identify targeting strategies
Source: Oncotarget. 2017 Feb 24;8(16):25848–63. doi: 10.18632/oncotarget.15667 (PMC5432221; doi:10.18632/oncotarget.15667)
Supplement: Supplementary file 1 [file oncotarget-08-25848-s001.pdf]

Inflammatory breast cancer tumor emboli express high levels of anti-apoptotic proteins: use of a quantitative high content and high-throughput 3D IBC spheroid assay to identify targeting strategies

SUPPLEMENTARY MATERIALS

SUPPLEMENTARY FIGURE

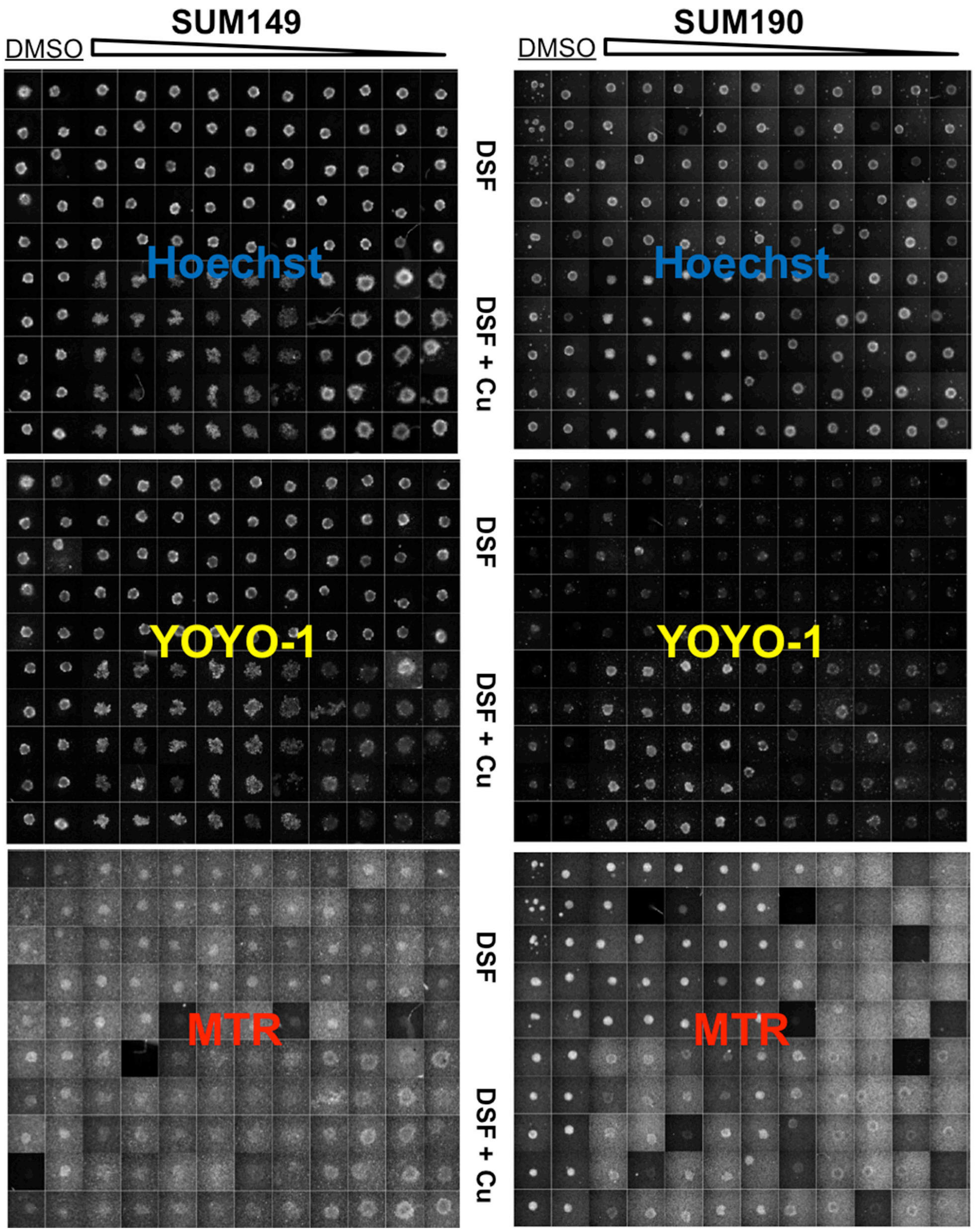

Supplementary Figure 1: Multi-well plate map for high content and high-throughput analysis. Representative images of the 384-well plate map for SUM149 and SUM190 shown to indicate the presence of single spheroid in each well with DMSO, DSF, Cu, or DSF-Cu treatments and corresponding dye used for the quantitative high content imaging analysis.
